# Criminalization Through Transinstitutionalization: A Critical Review of the Penrose Hypothesis in the Context of Compensation Imprisonment

**DOI:** 10.3389/fpsyt.2018.00534

**Published:** 2018-10-25

**Authors:** Sebastian Schildbach, Carola Schildbach

**Affiliations:** Praxisgemeinschaft für Psychosomatik, Gais, Switzerland

**Keywords:** compensation imprisonment, mental disorders, penrose hypothesis, transinstitutionalization, DIA-X

## Abstract

In 1939, the Penrose hypothesis suggested that the number of psychiatric hospital beds was inversely related to the size of prison populations. Central to a causal interpretation of the Penrose hypothesis is the idea that a small proportion of any population requires institutional mental care. Several studies re-examining longitudinal and cross-sectional data found that a fall in available psychiatric hospital beds occurred over the same period as a rise in prisoner numbers. The observed inverse relationship was mostly interpreted as being the consequence of a lack of compassion for the disadvantaged in society, while other studies concluded that the correlation was spurious and determined by confounders. In Germany, Austria, and Switzerland, lawbreakers who are unwilling or unable to pay a fine for committing a petty crime such can face compensation imprisonment. Every tenth German detainee serves compensation imprisonment with an average incarceration time of 2–3 months. We analyzed the social-economic backgrounds and the levels of mental disorders in four populations of compensation prisoners, consisting of 100 participants each, in the German capital Berlin in 1999, 2004, 2010, and 2017. Largely, the compensation prisoners were homeless, single, and unemployed, exhibited a high degree of substance abuse and showed an extraordinary high prevalence of mental disorders. Unfortunately, as the average stay in prison is short, there are no decisive concepts for social rehabilitation after imprisonment. In addition to a lack of resocialization, potential job loss, and social stigmatization, the newly acquired subcultural contacts facilitate reoffending. This study aims to give an overview of the medical, sociologic, and psychopathologic examinations on compensation prisoners. By analyzing trends in the prevalence of mental disorders, we will discuss the medical appropriateness and sociologic sense of compensation imprisonment with respect to the Penrose hypothesis. Thereby, we aim at shedding light on the question whether compensation imprisonment is an indispensable tool for law enforcement or if it is a punishment of the poor or mentally ill, which further deteriorates their unfavorable socio-economic situation. Finally, we will propose measures to reduce the number of reoffenders and to enable the compensation detainees to reintegrate successfully into society.

## Introduction

In 1939, the English scientist Lionel Penrose found an inverse correlation between the size of psychiatric inpatient clinics and the number of detainees based on cross-sectional data from diverse European countries (1). His assumption that the number of psychiatric hospital beds was inversely related to the size of prison populations was later termed the “Penrose hypothesis.” A common expression summarizing Penrose's findings is “transinstitutionalization,” which refers to a process where mentally ill individuals, who are discharged from, or no longer admitted to, mental hospitals, are frequently found in prisons (2–4).

Even 80 years after its formulation, the Penrose hypothesis has neither been rejected nor confirmed. Despite repeated observations of transinstitutionalization, and an increase of the numbers of imprisoners, it is still unclear whether there is an association between capacities in psychiatric clinics and prison sizes (5).

In 2004, a meta-analysis on data from 158 countries found the opposite relationship compared to Penrose, namely that in low-and-middle-income countries, prison, and psychiatric populations were positively correlated (6). However, similar to the preceding study by Penrose, this meta-analysis used cross-sectional data.

Longitudinal data on treatment histories of U.S. prisoners revealed that the decrease in the number of psychiatric hospital beds accounted only for a small proportion of the expanding prison populations between 1968 and 1978 (7). Longitudinal data from Europe indicated that psychiatric care might have reached a phase of transinstitutionalization (8, 9), in which the numbers of mental health care beds might further decline, and that at the same time, capacities in prisons might extend (10). Still, none of these studies provided undisputable evidence for a direct correlation between decreasing capacities of mental health care institutions beds and increasing prison populations (11, 12). Another study suggested that both the numbers of mental health care beds and the numbers of detainees might be influenced by economic factors (13). However, none of the published studies have thus far sufficiently disproved Penrose's direct inverse association theory (14).

A recent longitudinal study found that since 1990, capacities of mental health care institutions were considerably cut down in South America, while prison populations boosted despite a strong economic growth (15). The observed developments appear to support the Penrose hypothesis, because the numbers of psychiatric beds decreased more substantially when and where the number of imprisoners increased (15). Comparable conclusions of a decline of mental health care beds and a simultaneous rise in numbers of detainees were documented in Ireland and Norway (16, 17).

In Germany, Austria, and Switzerland, the penalty system includes a certain type of punishment termed “compensation imprisonment.” If a convicted person refuses to pay the fine for a crime, he or she must go to jail instead for a short period. This compensation imprisonment is regulated under section 43 of the German Penal Code and is conceived to ensure the effectiveness of the penalty system (18, 19). The proportion of compensation prisoners amounts for ~10% of all inmates in Germany (20). For nearly two decades, the meaningfulness of compensation imprisonment has been discussed (21–23). Because of the fact that the mean period of imprisonment is short, there are no meaningful approaches for social rehabilitation after imprisonment. As a consequence of this lack of resocialization, the detainees have to face potential job loss and social stigmatization, and the newly achieved contacts with other criminals facilitate reoffending (24).

In our previous longitudinal study from 1999 to 2017 on the prevalence of mental diseases in compensation prisoners, we found that 72.75% of these special clientele suffered from alcohol-induced mental and behavioral disorders, 45.5% suffered from mental and behavioral disorders due to use of illegal drugs, 35% exhibited phobic anxiety disorders, and 26.25% showed signs of depressive disorders (25). In addition, somatoform disorders and dysthymia were found at frequencies between 10 and 20% (25).

Therefore, our hypothesis is that compensation imprisonment is a punishment of the poor and mentally ill. With respect to the Penrose hypothesis, we suggest that the proposed process of transinstitutionalization can most likely be observed in compensation prisoners, as these detainees would most likely benefit from a mental health care treatment, while they are put into prison instead. Thereby, compensation imprisonment increases inequality and poverty among people at the edge of society.

## Study population and methods

### Study population

The process of data acquisition and diagnosis of mental disorders was described before (25). In total, four study populations of randomly selected compensation prisoners were collected in the years 1999 (26), 2004 (27), 2010 and 2017 (25). As all study participants were diagnosed with the same diagnostic system DIA-X, the data were pooled for inferring the prevalence of diverse mental disorders in compensation prisoners.

### Diagnostic system DIA-X

For diagnosing psychiatric disorders, the long form of the computer-aided expert system DIA-X was used (28). DIA-X supports the user reliably and efficiently with the diagnosis of about 100 mental disorders according to ICD-10 (International Classification of Diseases) and DSM-IV (Diagnostic and Statistical Manual of Mental Disorders) (29). The long version of DIA-X is a standardized interview for measuring mental disorders in the last 12 months. The modular structure and the possibilities of branching ensure that despite the standardization only the symptom constellation important for the respective subject is placed in the center of the interview. DIA-X was applied as computer version. For the DIA-X standardized interview, the interrater agreement was reported to range between 97 and 100% for the most common mental disorders, and the interrater kappa was reported to range between 0.67 (somatization disorder) and 0.99 (agoraphobia) (28).

Additionally, selected social and demographic characteristics basic data were collected. Each session lasted between 90 and 120 min, on average 105 min (25).

### Data sources

In order to compare compensation prisoners with general prisoners and with the general population in terms of the prevalence of the diverse mental disorders, a literature search was performed to assess the prevalence rates of the mental illnesses. For mental diseases in general prisoners, the following articles were consulted: (30–34).

The prevalence of mental diseases in the general population were extracted from Angst (35), Martin (36), Bloomfield et al. (37), Hilderink et al. (38), Patra and Sarkar (39), Qian et al. (40), Grant et al. (41), Chang et al. (42), Vandeleur et al. (43), and Leutgeb et al. (44).

In order to assess the percentage of compensation prisoners in all detainees and to be able to determine a temporal trend, we used data from the German Federal Statistical Office. The Federal Statistical Office publishes at regular intervals the stock of prisoners in the German prisons with respect to their regional placement and with respect to the form of imprisonment on the deadlines March 31, August 31, and November 30 of each year. For this study, the total number of inmates and the number of compensation prisoners were taken from the collections on November 30 each year starting from 2009 to 2017 (45, 46).

### Statistical analyses

A simple linear regression analysis was used for modeling the relationship between the percentage of compensation prisoners on all prisoners (dependent variable) and the time in years since 2009 (independent variable).

## Results

### Mental disorders in detainees, compensation detainees, and general population samples

Table 1 gives an overview of the prevalence of various mental illnesses among prisoners and the general population. Furthermore, a distinction was made between detainees in general and compensation prisoners.

**Table 1:** Overview of the average prevalence of mental disorders compensation prisoners, general prisoners and in the general population.

**ICD-10**	**Diagnosis**	**Prevalence in compensation prisoners (%)**	**Prevalence in general prisoners (References)**	**Prevalence in general population (References)**
F10	Mental and behavioral disorders due to use of alcohol	72.75	21–46.7% (31, 33, 34)	3–5% (37)
F11-16	Mental and behavioral disorders due to drug abuse	50.25	21–38% (30–34)	9.9% (41)
F20–F29	Schizophrenia, schizotypal, delusional, and other non-mood psychotic disorders	3.75	0.3–3.4% (30, 31, 47)	1.25–1.5% (36, 42)
F30	Hypomania	3.0	0.5% (32)	5.5% (35)
F32–F33	Depressive disorders	26.25	3.3–26.2% (31, 34, 47)	16.8–19.2% (43)
F34.1	Dysthymia	11.5	2.1–5.2% (32)	2.0–3.3% (36, 43)
F40	Phobic anxiety disorders	35	2.4–7.3% (32, 47)	6.2% (36)
F43	Reaction to severe stress, and adjustment disorders	7.0	1.9–4.6% (30, 31)	0.9% (39)
F45	Somatoform disorders	16	1.7% (47)	1.5–21.0% (38, 44)
F50	Eating disorders	2.25	0.3–2.0% (31)	1.01% (40)

The first striking feature of this statistic is that the prevalence of mental illness due to the use of alcohol among compensation prisoners was 72.75%, while prisoners in general exhibited prevalence rates of 21–47%. In the normal population, the prevalence of alcohol-related mental illnesses was only around 3–5%. Compensation prisoners were therefore three times as likely to suffer from alcohol-related mental illness as average prisoners were and 10–20 times as likely to be troubled by alcohol-related mental illness as the average population.

Mental illness caused by substance abuse was found to have a prevalence of 50.25% among compensation prisoners, while its prevalence varied between 21 and 38% among general inmates and lay at only 10% in the general population.

In hypomania and depressive disorders, there were no deviations in the prevalence in compensation prisoners. Dysthymia affected 11.5% of compensation prisoners but only 2.1–5.2% of average prisoners and 2.0–3.3% of the norm population. With regard to dysthymia, the prevalence of compensation prisoners was thus threefold higher than that of the average population.

Phobic anxiety disorders were detected in 35% of compensation prisoners, but the prevalence in the normal population was only 6.2%. The difference in adjustment disorders was particularly pronounced: with a prevalence of 7% for compensation prisoners and 1.9–4.6% for general detainees and only 0.9% for the norm population, the presence of adjustment disorders among compensation inmates exceeded the norm many times over.

Another eye-catching finding was that 16% of compensation inmates were diagnosed with somatoform disorders, while only 1.7% of other inmates and only 1.5–21.0% of the general population suffered from somatoform disorders.

On average, about 1% of the population suffers from eating disorders, with women being significantly more affected than men are. In prisons, on average, 0.3% of men and 2.0% of women suffered from eating disorders. Therefore, it was conspicuous that our study population of compensation prisoners, which consisted exclusively of men, had an eating disorder rate of 2.25%.

### Temporal development of numbers of prisoners in germany

Table 2 presents the development of the numbers of all detainees and of compensation detainees in Germany from 2009 to 2017. The number of inmates in Germany has declined considerably in recent years. From the beginning of available records in 2003 until 2009, the number of inmates exceeded 70,000 every yearly cut-off date (45). The number of inmates was below 70,000 for the first time in 2010 (45), and since then the numbers have been decreasing constantly (46). On the other hand, the numbers of compensation prisoners remained constant or increased steadily since 2009, both in absolute terms and in percentage terms.

**Table 2:** Number of prisoners in Germany.

**Year**	**Total number of prisoners**	**Compensation prisoners**
2009	70,817	3,868 (5.5 %)
2010	69,385	3,776 (5.4 %)
2011	68,099	3,802 (5.6 %)
2012	65,902	3,936 (6.0 %)
2013	62,632	3,968 (6.3 %)
2014	61,872	4,460 (7.2 %)
2015	61,737	4,135 (6.7 %)
2016	62,865	4,487 (7.1 %)
2017	64,351	4,580 (7.1 %)

*The numbers were collected at the end of November in each year*.

To sum up, the number of compensation prisoners in Germany, who were in jail by the end of November in each year, increased nationwide from 3,868 detainees in 2009 to 4,580 detainees in 2017, with a simultaneous decrease in the total number of prisoners. While 5.5% of all inmates were compensation prisoners in 2009, in 2017 the amount of compensation prisoners increased to 7.1% of all prisoners. More concretely, this finding in relative terms meant that the total number of detainees decreased by 9.1% from 2009 to 2017, while the number of compensation prisoners increased by 18.4% between 2009 and 2017.

A simple linear regression analysis with the time in years as independent factor and the percentage of compensation prisoners on all prisoners as dependent variable explained a large amount of variance in the data (*R*^2^ = 0.871). Every year, the proportion of compensation prisoners on all prisoners increased by 0.253%, and the association was highly significant (*p* < 0.001).

## Discussion

### Mental disorders in compensation detainees

Convicts who cannot pay the fine for committing a petty crime like fare evasion have to serve compensation imprisonment. The risk of compensation imprisonment is therefore many times greater for poor people than for financially well-off people.

The comparison of the prevalence of mental disease in compensation prisoners with population samples from general prisons and from the general population yielded a clear result: compensation prisoners are many more times more prone to suffer from mental diseases induced by alcohol and drug abuse than the normal population. Even in comparison with population samples from worldwide prisons, the prevalence of alcohol-and drug-abuse related disorders was extraordinarily high. Our finding that 72.75% of compensation prisoners suffered from mental and behavioral disorders due to use of alcohol is in line with the findings of Konrad and Opitz-Welke, who reported that 77% of their study population consisting of compensation and investigation prisoners were diagnosed with alcohol abuse (48)

In addition, dysthymia, phobic anxiety disorders, adjustment disorders, somatoform disorders, and eating disorders occurred at frequencies wide above the standard levels. The exceptionally high prevalence of adjustment disorders could reflect immediate negative reactions to incarceration (49).

One explanation for this observation could be an interaction between being poor and being mentally ill. Indeed, several studies could demonstrate that people who live in poverty appear to be at higher risk for mental illnesses (50–52). However, the association between poverty and mental disorders is complex and bidirectional. On the one hand, besides genetics, adverse life events or substance abuse, poverty can be a main factor causing mental illness. On the other hand, mental illness may lead people down a road to poverty, because of disability, stigma or the need to spend extra money on health care (50, 51). Lund and colleagues suggest that poverty more often leads to depression while disorders like schizophrenia more often lead to poverty (50, 51).

Therefore, the conversion of the monetary fine for committing a petty crime into imprisonment primarily affects the socially marginalized, the poor, and the mentally ill. Consequently, compensation imprisonment may constitute the backbone of the sanction system, but it seems dysfunctional to our subjects.

### Results in relation the penrose hypothesis

The longitudinal analysis of prisoner numbers in Germany yielded a clear trend: while the number of people in jail is constantly decreasing, the number of compensation prisoners is constantly increasing. As social-demographic study on compensation prisoners demonstrated that these people were mainly homeless, unemployed, and had hardly any sustaining family background (25). The finding that compensation prisoners suffered from a wide spectrum of mental disorders, which exceeded the standard population by a magnitude, underscored the hypothesis that these people are in fact victims of a transinstitutionalization process.

If prisons in fact could be a substitute for mental health care clinics, then the question arises to what these facilities could offer to the many inmates with serious mental disorders. One modality that jails offer is structure, which is implemented in the form of a protected setting and of employees who can hold back improper and destructive behavior, and conceive a personalized psychiatric treatment regime. However, for those people with serious mental disorders and who serve compensation imprisonment, this structured setting is not sufficient, as their stay in prison is generally very short and standard treatment plans are not being carried out for reasons of time. For this clientele, psychiatric inpatient treatment and drug and alcohol withdrawal would be preferable to incarceration into a prison.

It is broadly accepted that numerous people with genuine psychological problems, who have been criminalized, could be dealt with effectively in the community, if there were sufficient and available treatment facilities (53). However, in Eastern Germany, after the political change, the number of general psychiatric beds fell by 61% and the prisoners' rate dropped by 77%, so that within a few years the rates between East and West Germany converged. In both parts of Germany, capacities were built up in the execution of sentences, assisted living and rehabilitation facilities. In West Germany, the number of psychiatric beds fell by 40% between 1989 and 2003 (54). However, at the same time, forensic psychiatric bed numbers increased in most countries, especially in East Germany (12). Consistent with the Penrose hypothesis, it seemed that the extensive decline in general mental health care beds might have partly been compensated by a rise in forensic mental health care beds. Although the reduction of general psychiatric beds may not have caused a growth of prison populations, available data do not allow excluding a possible transinstitutionalization of people with mental disorders from psychiatric hospitals to prisons (12).

Lamentably, the deficient treatment of mentally challenged people during compensation imprisonment and the inadequate number of clinic beds (acute, intermediate, and long term) for the individuals who require them are some of the realities of transinstitutionalization that have set the stage for criminalization (55).

The Penrose hypothesis has been a valuable reference point for investigations into the intricate relationship between the mental health care system and the legal enforcement system for more nearly 80 years (53). Our results do not prove that validity of the Penrose hypothesis, but in the special setting of compensation imprisonment, our observations support the idea of a transinstitutionalization process. This transinstitutionalization process could possibly lead to an unintentional stigmatization of socially marginalized, poor and mentally ill persons as criminals (56, 57).

However, it is important to emphasize that within the context of this study, the Penrose hypothesis was used as an analogy and that our results were purely descriptive. Therefore, our implications and conclusions are of speculative nature and cannot be confirmed by the descriptive data.

### Limitations

One limitation of the study is that was not possible to diagnose personality orders with the diagnostic system DIA-X. However, antisocial, borderline, and paranoid personality disorders were associated strongly with substance-use disorders (58–60). Therefore, determining the prevalence of personality disorders would provide interesting insights into the mental health of this particular study population, which has an extremely high rate of substance abuse. The diagnosis of personality disorders could be a relevant factor especially for compensation prisoners minimize recidivism among those in legally supervised treatment (61).

Another limitation of using the diagnostic system DIA-X is constituted by a potential underestimation of more chronic forms of schizophrenia that are dominated by negative symptoms in compensation prisoners.

Finally, we cannot validate whether the reduction of mental health care beds in Germany concerned mostly chronic diseases like schizophrenia or mental disabilities, as there are no statistical reports on this issue. However, this information would be a prerequisite in order to prove that substance abusers were especially affected by the reduction of psychiatric hospital beds.

### Recommendations

The German law already offers an alternative to compensation imprisonment, which is community service. People sentenced to serve compensation imprisonment can apply for serving voluntary community work instead (19). This seems very meaningful, as imprisonment would further deteriorate their precarious financial and social situation and would further impair their fragile state of mental health. If these people are put to jail and released again without support, they will find themselves in a vicious circle without the hope of ever escaping their compromising situation.

Given that a large proportion of the compensation prisoners suffered from mental illness, we believe that it is advisable to first psychologically diagnose anyone convicted of compensation imprisonment. This could be achieved with the DIA-X diagnostic system, for example. Then a therapy should take place accompanying the voluntary work, which should deal with the respective problems of the individual. For serious mental illness, a transfer to a psychiatric hospital would be worth considering. In any case, nothing should be left unturned to integrate the convict into a functioning social environment.

## Conclusions

Our studies add weight to claims that compensation imprisonment leads to an ethically questionable and clinically inappropriate transinstitutionalization and further criminalization of poor or mentally ill people from the edge of society into prisons, which are poorly set up to treat and support them. Policymakers should therefore consider the current limits of compensation imprisonment.

## Ethics statement

In January 2016, a comprehensive research proposal was submitted to the criminal services of the penal institutions in Berlin and to the social services of the penal institution Plötzensee, which were both approved in February 2016. In addition, the prison management of the penal institution Plötzensee approved the study in April 2016. Finally, the Berlin Commissioner for Data Protection issued a clearance certificate in May 2016.

## Author contributions

CS conducted the literature search, collected and evaluated the data, performed the statistical analyses, and supported SS with writing the article wrote the article. SS conceived and prepared the study, wrote the research proposal, communicated with the authorities, recruited the patients, organized the data collection, and wrote the article.

### Conflict of interest statement

The authors declare that the research was conducted in the absence of any commercial or financial relationships that could be construed as a potential conflict of interest.
